# Sodium-glucose Cotransporter-2 Induced Diabetic Ketoacidosis with Minimal Hyperglycemia

**DOI:** 10.5811/cpcem.2017.12.36213

**Published:** 2018-01-11

**Authors:** D. Taylor Gammons, Francis L. Counselman

**Affiliations:** * Eastern Virginia Medical School, Department of Emergency Medicine, Norfolk, Virginia; † Emergency Physicians of Tidewater, Norfolk, Virginia

## Abstract

The case of a 56-year-old man with a history of type 2 diabetes mellitus who presented to the emergency department in diabetic ketoacidosis (DKA) with only mild hyperglycemia is presented. The patient was taking empagliflozin (Jardiance®), a sodium-glucose cotransporter -2 inhibitor, which has now been recognized as causing this unusual presentation of DKA. Emergency physicians need to be aware of this complication, as the euglycemia/mild hyperglycemia and a history of type 2 diabetes mellitus can make the correct diagnosis of DKA a challenge.

## INTRODUCTION

We present the case of a 56-year-old man with a history of type 2 diabetes mellitus who presented to the emergency department in diabetic ketoacidosis (DKA) with only a slightly elevated serum glucose. The patient was taking empagliflozin (Jardiance®), a sodium-glucose cotransporter-2 inhibitor. There are increasing reports of this unusual complication in patients taking this class of medication. Emergency physicians need to be aware of this complication, as the euglycemia and history of type 2 diabetes mellitus can make the correct diagnosis of DKA challenging.

## CASE PRESENTATION

A 56-year-old man presented to the emergency department (ED) with a five-day history of left upper quadrant abdominal pain and low-grade fever. The patient described the pain as achy, constant and worse with eating. The patient denied nausea, vomiting or diarrhea. There was no history of trauma or similar symptoms in the past. The past medical history was significant for coronary artery disease (requiring two stents), type 2 diabetes mellitus, hypertension and hypertriglyceridemia. The patient stated he was compliant with his medications, including losartan-hydrochlorothiazide 100/12.5 mg, metoprolol 50 mg, glipizide XL 10 mg, empagliflozin (Jardiance®) 25 mg, sitagliptin-metformin 500/1000 mg, prasugrel 10 mg, rosuvastatin 10 mg, and aspirin 81mg. He denied smoking cigarettes, but admitted to several drinks at “happy hour.” He denied any alcohol use in the previous five days.

The vital signs were heart rate 97 beats per minute; respiratory rate 18 breaths per minute; blood pressure 196/96 mm Hg; temperature of 99.2 °F (37.3 °C); and 94% oxygen saturation on room air. The patient appeared in no distress. The head, eyes, ears, nose and throat exam was normal, as were the heart and lung exams. The abdomen was soft, with mild tenderness in the epigastrum and left upper quadrant, without guarding or rebound. The remainder of the physical exam, including the extremities and neurologic, were normal.

The emergency physician (EP) ordered an electrocardiogram (ECG), complete blood count (CBC), basic metabolic profile (BMP), lipase, urinalysis and troponin T. The CBC was normal, as was the lipase. The BMP was remarkable for a glucose of 142 mg/dL, sodium of 128 mmol/L, chloride of 87 mmol and a bicarbonate of 19 mmol/L. The blood urea nitrogen, creatinine and potassium were normal. The urinalysis was remarkable for 80 mg/dL of ketones and greater than 500 mg/dL of glucose. The ECG revealed only non-specific ST and T-wave changes in the lateral leads. The patient’s anion gap was markedly elevated at 21.7. After reviewing the laboratory results, the EP was concerned about the large anion gap metabolic acidosis. Additional studies ordered included a serum acetone, beta-hydroxybutyrate and a lactic acid.

The EP ordered one liter of normal saline intravenous (IV) bolus and morphine 4 mg and ondensetron 4 mg IV for the abdominal pain. The EP went back and specifically asked the patient about possible causes of the anion gap metabolic acidosis, including ethylene glycol, propylene glycol or methanol ingestion, alcohol abuse, iron or isoniazid use, excessive salicylate use, and dieting (starvation). The patient denied all of these. The serum acetone was reported as “moderate,” the beta-hydroxybutyrate was elevated at 47.6 mg/dL (normal range 0.2 – 2.8 mg/dL) and a serum lactate of 1 mmol/L (normal range 0.5 – 2.2 mmol/L). A repeat BMP revealed the bicarbonate had decreased to 17 mmol/L.

The EP thought the patient was in diabetic ketoacidosis (DKA), but was confused by the only slightly elevated blood glucose and the fact that the patient was a type 2 diabetic. The EP performed a quick literature search and found that diabetic patients on sodium-glucose cotransporter-2 (SGLT2) inhibitors were at risk for DKA with euglycemia. This patient was on just such a medication (empagliflozin) and had been so for the preceding three years. The patient was admitted to the medicine service and started on an IV insulin drip with concomitant IV dextrose 5% in normal saline drip at 125 cc/hr.

A computed tomography (CT) scan of the abdomen/pelvis with IV contrast was ordered to further evaluate the cause of the left upper quadrant abdominal pain. The CT demonstrated “findings of acute pancreatitis with confluent infiltrative phlegmon around the tail and left side of the pancreatic body, extending to lower portion of the spleen and to left anterior and lateral pararenal space. There was no definable pancreatic mass.” Gastroenterology (GI) was consulted and thought the patient had acute chemical pancreatitis secondary to the DKA and his underlying hypertriglyceridemia. Endocrinology was consulted; they felt the DKA was most likely due to the empagliflozin and that the patient needed to avoid this medication in the future. After four days on an insulin drip, the anion gap decreased to 14, there was no definable serum acetone, and the beta-hydroxybutyrate level returned to normal. The patient could then be weaned off the insulin drip and started on medications by mouth. The patient was discharged home on day five. The patient was instructed to stop taking the empagliflozin and to avoid all such similar medications in the future. His abdominal pain was much improved and he was eating a full diet. The patient was scheduled to follow up with GI and endocrinology in one week.

## DISCUSSION

Diabetic ketoacidosis is a life-threatening complication of diabetes mellitus. It usually occurs in type 1 diabetics, but may occur in type 2 patients under moderate to severe physiologic stress. According to the American Diabetes Association, the diagnostic criteria for DKA includes a plasma glucose greater than 250 mg/dL, positive serum or urinary ketones, an arterial pH of less than 7.3, serum bicarbonate less than 18 mEq/L and a high anion gap. The key diagnostic feature is the elevated circulating total blood ketone concentration.^1^

Euglycemic diabetic ketoacidosis is now defined as diabetic ketoacidosis with a blood glucose concentration of less than 200 mg/dL, and occurring primarily in patients with type 1 diabetes.^2^ Euglycemic DKA has been described in the past but is considered rare.^3^,^4^ Munro et al. described the first case series of euglycemic DKA in 1973, consisting of 37 episodes in 17 patients.^5^ They suggested that in type 1 diabetics who were unable to maintain sufficient carbohydrate intake, but maintained hydration status and continued their normal insulin intake, could develop severe ketoacidosis without pronounced hyperglycemia.^5^ Thawabi et al. presented two cases of euglycemic DKA in type 1 diabetic patients that were consistent with Munro’s physiologic explanation.^1^ One case was precipitated by an underlying infection; the other by acute pancreatitis.^1^

CPC-EM Capsule

What do we already know about this clinical entity?

Diabetic ketoacidosis (DKA) is a life-threatening complication of diabetes mellitus. It usually occurs in patients with type 1 diabetes, and is associated with a blood glucose level of greater than 250mg/dL, a serum bicarbonate level less than 15 mEQ/dL, and a pH less than 7.3.

What makes this presentation of disease reportable?

With the introduction of sodium-glucose co-transporter-2 inhibitor medications for the treatment of type 2 diabetes mellitus, this classic presentation of DKA no longer holds true.

What is the major learning point?

These medications can cause DKA in a type 2 diabetic patient with euglycemia or only mild hyperglycemia.

How might this improve emergency medicine practice?

Emergency physicians must be aware of this atypical presentation of DKA to ensure making the correct diagnosis.

More recently, there have been several reports of euglycemic DKA attributed to the use of SGLT2 inhibitors.^6^,^7^ Roach et al. described the first case of euglycemic DKA associated with the use of empagliflozin, the same medication as in our patient.^6^ The case involved a 64-year-old woman with type 2 diabetes who developed DKA following five days of empagliflozin use.^6^ The other SGLT2 inhibitors – canagliflozin and dapagliflozin -- have all been associated with euglycemic DKA.^3^,^7^,^8^ In fact, in May 2015 the U.S. Food and Drug Administration (FDA) issued a warning that the “SGLT2 inhibitors can cause too much acid in the blood,” and that patients should stop taking their SGLT2 inhibitor and seek medical attention immediately if they develop symptoms of ketoacidosis.^9^ This warning was based in part because of the FDA identifying 73 cases of ketoacidosis requiring hospitalization in patients on SGLT2 inhibitors between March 2013 and June 2015.^3^,^9^ The median time to onset of symptoms was 43 days, with a range of 1 to 365 days.^3^,^9^

The SGLT2 inhibitors are one of the newest class of oral hypoglycemic medications used to treat diabetes mellitus. These drugs are FDA approved for use in adults with type 2 diabetes. Canagliflozin was the first to be introduced, followed by dapagliflozin and empagliflozin.^3^ These medications are available as single-ingredient products, or in combination, usually with metformin. These drugs have a novel mechanism of action by inhibiting the enzyme SGLT located in the proximal renal tubules. This enzyme is responsible for reabsorbing approximately 90% of glucose found in the filtrate within the kidney.^10^ By blocking this enzyme, the blood glucose is decreased because of an increase in renal glucose excretion. These drugs have a relatively pronounced blood glucose lowering effect, with a low risk for hypoglycemia when administered as monotherapy.^2^ Given that these drugs promote excretion of glucose (an energy source) into the urine, treatment with these drugs also reduces body weight and have pleiotropic effects attributable to weight loss, including amelioration of insulin resistance, dyslipidemia, and nonalcoholic fatty liver disease.^2^ In addition, empagliflozin was shown to reduce cardiovascular and all-cause mortality, and reduce hospitalization for heart failure, compared to placebo in over 7,000 adult patients with type 2 diabetes mellitus.^11^

These drugs, however, are not without adverse effects. In addition to ketoacidosis, they are associated with urinary tract infections (in some cases, leading to urosepsis) and vaginal candidiasis. However, it is the ketoacidosis, and specifically the euglycemic ketoacidosis, that is the most concerning. There are several proposed theories to explain the link between SGLT2 inhibitors and ketoacidosis. One possible mechanism involves a decreased secretion of insulin from pancreatic cells in response to the lowering of blood glucose via urinary excretion.^4^ This results in decreased circulating insulin and its antilipolytic activity, leading to increased free fatty acid production.^4^ One animal study suggests that SGLT2 inhibitors stimulate the secretion of the counterregulating hormone glucagon, which in turn contributes to the overproduction of ketone bodies.^3^,^8^,^12^ Finally, another animal study suggests that SGLT inhibitors might decrease the renal clearance of ketone bodies.^3^,^4^ The net result is a stimulation of the ketogenesis pathway and an increase in serum ketones, which predispose the body to ketoacidosis. This effect is compounded in the presence of physiologic stressors, such as starvation or dehydration.^3^

## CONCLUSION

Although the reason behind SGLT2 inhibitors causing ketoacidosis, often euglycemic ketoacidosis, might not be fully understood, the association definitely exists. Emergency physicians must recognize that diabetic ketoacidosis can develop in type 2 diabetic patients with normal or only slightly elevated blood glucose levels in patients taking SGLT2 inhibitors.

## References

[1] Thawabi M, Studyvin S (2015). Euglycemic diabetic ketoacidosis, a misleading presentation of diabetic ketoacidosis. N Am J Med Sci.

[2] Ogawa W, Sakaguci K (2016). Euglycemic diabetic ketoacidosis induced by SGLT2 inhibitors: possible mechanism and contributing factors. J Diabetes Invest.

[3] Jazi M, Porfiris G (2016). Euglycemic diabetic ketoacidosis in type 2 diabetes treated with a sodium-glucose cotransporter-2 inhibitor. Can Fam Physician.

[4] Simeon IT, Blau JE, Kristina IR (2015). SGLT2 inhibitors may predispose to ketoacidosis. J Clin Endocrinol Metab.

[5] Munro JF, Campbell IW, McCuish AC (1973). Euglycaemic diabetic ketoacidosis. Br Med J.

[6] Roach P, Skiercynski P (2016). Euglycemic diabetic ketoacidosis in a patient with type 2 diabetes after treatment with empagliflozin. Diabetes Care.

[7] Fralick M, Schneeweiss S, Paterno E (2017). Risk of diabetic ketoacidosis after initiation of an SGLT2 inhibitor. N Engl J Med.

[8] Hayami T, Kato Y, Kamiya H (2015). Case of ketoacidosis by a sodium-glucose cotransporter 2 inhibitor in a diabetic patient with a low-carbohydrate diet. J Diabetes Invest.

[9] US Food and Drug Administration (website) (2015). FDA Drug Safety Communication: FDA revises labels of SGLT2 inhibitors for diabetes to include warning about too much acid in the blood and serious urinary tract infections.

[10] Kim GW, Chung SH (2014). Clinical implication of SGLT2 inhibitors in type 2 diabetes. Arch Pharm Res.

[11] Zinman B, Wanner C, Lachin JM (2015). Empagliflozin, cardiovascular outcomes, and morbidity in type 2 diabetes mellitus. N Engl J Med.

[12] Kibbey RG (2015). SGLT-2 inhibition and glucagon: cause for alarm?. Trends Endocrinol Metab.

